# Relationship between Lower Urinary Tract Dysfunction and Clinical Features in Chinese Parkinson's Disease Patients

**DOI:** 10.1155/2019/6820937

**Published:** 2019-03-05

**Authors:** Duo Xu, Shunchang Han, Jue Wang, Juan Feng

**Affiliations:** Department of Neurology, Shengjing Hospital, Affiliated Hospital of China Medical University, No. 36 Sanhao Street, Shen Yang 110004, China

## Abstract

**Background:**

Lower urinary tract (LUT) dysfunction is very common in Parkinson's disease (PD) patients. However, the number of studies conducted on LUT dysfunction and its related factors in Chinese PD patients is very limited, and there is no international consensus concerning the results.

**Methods:**

This cross-sectional study enrolled 100 Chinese PD patients. The patients were classified based on their overactive bladder symptom score (OABSS) and then assigned to either a PD with overactive bladder (PD-OAB) group or a PD with no overactive bladder (PD-NOAB) group. A binary logistic regression analysis was performed to identify the accompanying factors for overactive bladder (OAB). Next, correlations between the OABSS and patient sex, age, age of onset, disease duration, MDS-UPDRS-III, H-Y stage, PD subtype, treatment, education, and nonmotor symptoms were analyzed to identify factors correlated with LUT dysfunction.

**Results:**

Eighty nine (89%) of the PD patients suffered from LUT dysfunction, and OAB was diagnosed in 45 (45%) of those PD patients. The most common lower urinary tract (LUT) symptom in the PD patients was nighttime frequency (86%), followed by urgency (50%), urge incontinence (34%), and daytime frequency (17%). Patients in the PD-OAB group had an older age and age of onset, were at a more advanced Hoehn–Yahr stage, and had more severe motor symptoms and nonmotor symptoms, including worse cognition, and a greater incidence of REM sleep behavior disorder (RBD). A binary logistic regression analysis showed that a lower Frontal Assessment Battery (FAB) score, higher H-Y stage, and RBD accompanied with a higher prevalence of OAB in PD patients. A multiple linear regression analysis showed that the OABSS was significantly influenced by the FAB score, H-Y stage, RBD, and age.

**Conclusions:**

The FAB score, H-Y stage, and RBD are accompanying factors for OAB. A higher OABSS in PD patients was related to a lower FAB score for frontal lobe executive dysfunction, a higher H-Y stage for severity of motor disorders, RBD, and an older age.

## 1. Introduction

Parkinson's disease (PD) is the second most prevalent neurodegenerative disease and is caused by a loss of dopaminergic neurons in the substantia nigra. PD is characterized by the manifestation of motor symptoms such as bradykinesia, static tremor, and rigidity. In addition to motor symptoms, nonmotor symptoms such as hyposmia, sleep disorders, neuropsychiatric symptoms, and autonomic dysfunction become increasingly recognizable over time. These nonmotor symptoms accelerate disease progression and severely affect the patient's quality of life. Lower urinary tract (LUT) dysfunction is a common nonmotor symptom of PD and was recently reported to occur in 27%–63.9% of PD patients during any stage of their disease [1]. LUT dysfunction primarily presents with storage and voiding symptoms. A storage symptom can manifest as nighttime frequency, daytime frequency, urgency, or urge incontinence, while a voiding symptom can manifest as dysuria or a prolonged time of micturition. LUT dysfunction in PD patients manifests primarily with symptoms of storage difficulty, suggesting an overactive bladder (OAB).

Micturition is a complicated process controlled by the frontal lobe, basal ganglia, brainstem, spinal cord, and the other central autonomic networks [2, 3]. Some studies have found that LUT dysfunction can exacerbate the extent of cognitive dysfunction [4, 5] and might be an early sign of accelerating PD progression [6]. Therefore, discovering the relevant factors and using them to develop an early intervention strategy for LUT dysfunction in PD patients is of great importance. Here, we aim to identify the independent accompanying factors for OAB and the clinical factors those may aggravate the LUT dysfunction in Chinese PD patients.

## 2. Subjects and Methods

From May 2017 to March 2018, this cross-sectional study enrolled 100 PD patients who visited the Department of Neurology at Shengjing Hospital of China Medical University. PD was diagnosed according to the United Kingdom PD Society Brain Bank Clinical Diagnostic Criteria for PD [7]. No patient had any other types of neurological disease, severe urinary disorder, or secondary parkinsonian syndrome. Patients taking an anticholinergic drug were excluded from the study. Our study has been approved by the ethics committee and has therefore been performed in accordance with the ethical standards laid down in the 1964 Declaration of Helsinki and its later amendments. Each patient gave their informed consent prior to their participation in this study.

All patients were evaluated during the “on” state of PD. A physician specializing in movement disorders collected demographic and clinical data, including the patient's sex, age, age of onset, disease duration, years of education, levodopa equivalent daily doses (LEDD), LUT symptoms, motor symptoms, and nonmotor symptoms (hyposmia, RBD, constipation, anxiety and depression, and hallucination) during face-to-face interviews. Motor disability and disease severity were rated using the Hoehn–Yahr stage (H-Y stage) [8] and Movement Disorder Society Unified Parkinson's Disease Rating Scale III (MDS-UPDRS-III) [9], respectively. The enrolled patients were characterized by two subtypes, tremor-dominant and akinetic/rigid-dominant, based upon the initial motor symptoms and the most prevalent symptom during a standard neurological examination [10]. The patient's LUT disorder was evaluated using the overactive bladder symptom score (OABSS) [11, 12]. The nonmotor symptoms were evaluated by using the NMS questionnaire [13]. Global cognitive function was assessed by the Mini-Mental Status Examination (MMSE) [14], Montreal Cognitive Assessment (MoCA) [15], and Frontal Assessment Battery (FAB) [16].

The overactive bladder symptom score (OABSS) was developed by Homma et al. and has been validated as a simple and effective research tool for assessing an OAB [11, 12]. The scale has scores for four specific symptoms: daytime frequency, nighttime frequency, urgency, and urge incontinence. The maximal scores for the four symptoms are 2, 3, 5, and 5, respectively. Any symptom with a score ≥ 1 is considered to be due to LUT dysfunction, and a total OABSS ≥ 3 with an urgency score ≥ 2 indicates an OAB. Here, we used OABSSs to identify factors correlated with LUT dysfunction in a cohort of PD patients in China.

Based on the questions of the NMS questionnaire, “If you have loss or change in your ability to taste or smell,” “If you have talking or moving about in your sleep as if you are “acting” out a dream,” “If you have constipation (less than 3 bowel movements a week) or having to strain to pass a stool (faeces),” “If you have feeling anxious, frightened or panicky,” “If you have feeling sad, “low” or “blue,”” and “If you have seeing or hearing things that you know or are told are not there,” the patients who answer “yes,” respectively, are considered accompanying with hyposmia, RBD, constipation, anxiety, depression, and hallucination.

### 2.1. Statistical Analyses

All data were analyzed using the Statistical Package for the Social Sciences (SPSS), Version 17 (Chicago, IL, USA). The Kolmgorow–Smimov test was used to evaluate data for its normal distribution. Data with a normal distribution are presented as the mean ± standard deviation (*x* ± *S*), and the independent sample *t*-test was used for comparisons between two groups. Nonnormally distributed data are shown as the median value and a quartile range {*M* (*Q*25, *Q*75)}, and the Mann–Whitney *U* test was used for comparing the two groups. Count data are expressed as a frequency or percentage (%), and the chi-square test was used to compare the two groups. A binary logistic regression analysis was performed to identify the independent accompanying factors for OAB, and a multiple linear regression analysis was performed to evaluate the relation between the OABSS score and other independent variables. *P* values < 0.05 were considered statistically significant.

## 3. Results

### 3.1. Basic Information for the Recruited PD Patients

Our cohort of PD patients included 55 (55%) males with a mean age of 66.40 ± 9.30 years and 45 (45%) females with a mean age of 65.44 ± 6.81 years. There was no significant difference between the male and female patients in terms of mean age (*t*-test, *P*=0.555) or disease duration (Mann–Whitney *U* test, *P*=0.370). The numbers of PD patients with OAB (29 male patients vs. 16 female patients) and LUT dysfunction (52 male patients vs. 37 female patients) in the two groups were also similar (*P*=0.086 and *P*=0.061, respectively).

**Figure 1:**
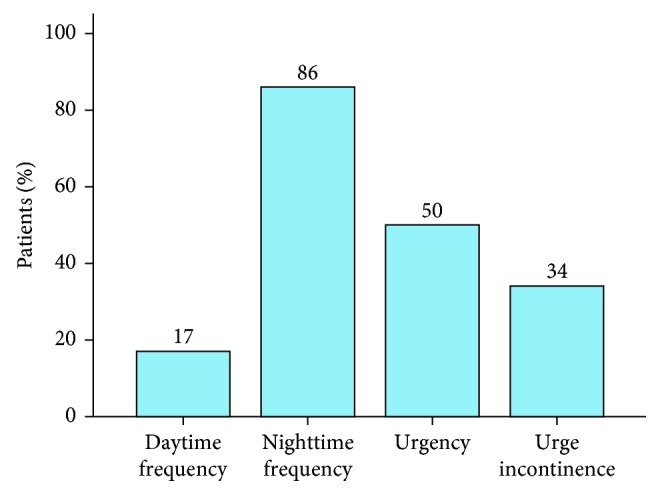
Frequency of urinary symptoms in PD patients.

Among the 100 participants, 89 (89%) PD patients suffered from LUT symptoms and 45 (45%) PD patients suffered from OAB. Nighttime frequency was the most common LUT symptom with a prevalence of 86%, followed by urgency (50%), urge incontinence (34%), and daytime frequency (17%) (Figure 1).

### 3.2. Comparison of Demographic and Clinical Characteristics of the PD-OAB and PD-NOAB Groups

The demographic and clinical characteristics of the enrolled PD patients are shown in Table 1. We divided all the enrolled patients into two groups: a PD-OAB group (an OABSS ≥ 3 with an urgency score ≥ 2) and a PD-NOAB group (an OABSS < 3 or an urgency score < 2). A comparison of the demographic and clinical characteristics of the two groups is also shown in Table 1.

There was no significant difference between the two groups in terms of gender (*P*=0.086), disease duration (*P*=0.325), education (*P*=0.439), LEDD (*P*=0.728), tremor-dominant subtype (*P*=0.949), akinetic/rigid-dominant subtype (*P*=0.935), hyposmia (*P*=0.550), constipation (*P*=0.788), anxiety and depression (*P*=0.614), hallucination (*P*=0.536), and MMSE score (*P*=0.146). However, the values for patient's age (*P* < 0.001), age of onset (*P*=0.002), Hoehn–Yahr stage (*P*=0.004), MDS-UPDRS-III score (*P*=0.039), and RBD (*P*=0.046) in the PD-OAB group were significantly higher than those in the PD-OAB group. Moreover, the MoCA and FAB scores in the PD-OAB group were significantly lower than those in the PD-NOAB group (*P*=0.007 and *P* < 0.001, respectively).

### 3.3. Independent Accompanying Factors for OAB

The accompanying factors found to be associated with OAB are shown in Table 2. A logistic regression model was used to identify factors potentially correlated with OAB. Variables that showed possible correlations with OAB in the univariate analysis (i.e., age, age of onset, Hoehn–Yahr stage, MDS-UPDRS-III score, RBD, MoCA score, and FAB score) were further analyzed in a logistic regression analysis. The stepwise model indicated that a higher Hoehn–Yahr stage (OR = 3.007, 95% CI = 1.373–6.585, and *P*=0.006), RBD (OR = 3.414, 95% CI = 1.196–9.745, and *P*=0.022) and a lower FAB score (OR = 3.344, 95% CI = 1.842–6.074, and *P* < 0.001) were accompanying factors for OAB in PD patients.

### 3.4. Factors That Could Independently Affect the Severity of Storage Dysfunction

A multiple linear regression analysis was performed to remove the confounding factors and identify factors that could independently affect storage dysfunction. The results showed that sex (*P*=0.071), age of onset (*P*=0.713), disease duration (*P*=0.482), tremor-dominant subtype (*P*=0.630), akinetic/rigid-dominant subtype (*P*=0.827), the MDS-UPDRS-III score (*P*=0.089), hyposmia (*P*=0.256), constipation (*P*=0.104), anxiety and depression (*P*=0.399), hallucination (*P*=0.243), the MMSE score (*P*=0.596), MoCA score (*P*=0.827), education (*P*=0.249), and LEDD (*P*=0.119) were all confounding factors. Furthermore, the OABSS was significantly influenced by the FAB score, H-Y stage, RBD, and age (*R* ^2^ = 0.428; FAB score, *P* < 0.001; H-Y stage, *P*=0.028; RBD, *P*=0.012; and age, *P*=0.004), indicating that PD patients with lower FAB scores, a higher H-Y stage, RBD, or of an older age had worse OABSSs than younger or less-affected patients (Table 3).

## 4. Discussion

LUT dysfunction in PD has received increasing attention in the recent years due to our increased knowledge of nonmotor symptoms. We found no gender-related differences in the average age, disease duration, and incidence of OAB and LUT dysfunction among the PD patients we recruited, which proves that the data we collected were logical. In our study, the most common LUT symptom in PD patients was nighttime frequency (86%), followed by urgency (50%), urge incontinence (34%), and daytime frequency (17%). The incidence of each of those symptoms was in accordance with those reported in previous studies, and all of them can seriously affect a patient's quality of life. The most striking finding in our study was that frontal lobe executive impairment, a severe disease stage, and RBD accompanied with a higher prevalence of OAB in PD patients. Furthermore, the severity of LUT disorders in PD patients was independently influenced by the above three factors and also old age.

According to the six pathological stages of Parkinson's disease proposed by Braak et al. [17], *α*-synuclein deposition begins in the medulla, gradually progresses to the pons and midbrain, and finally to the diencephalon and cortex. The early stage of PD cognitive dysfunction is mainly characterized by decreased executive function, which is closely related to prefrontal function, which includes functional areas such as task preparation, initiation, planning, and transformation. During this time period, it can be difficult for a clinician to identify any obvious cognitive decline in a patient [18]. However, as PD progresses, the risk for developing Parkinson's disease dementia also increases. The prefrontal cortex is thought to be the advanced center of the micturition reflex. When PD patients develop OAB, *α*-synuclein has already been deposited in the neocortex and prefrontal cortex in the extracellular area of the substantia nigra, corresponding to stage 4 or 5 of the Braak hypothesis. Hence, OAB symptoms can be a warning of progression to Parkinson's disease dementia. Therefore, it is important to explore the possible relationship between prefrontal function and OAB.

Our study used the FAB scale to detect the frontal lobe executive function and found that the FAB scores in the PD-OAB group were significantly lower than those in the PD-NOAB group. We also found that the OABSS was negatively correlated with the FAB score in PD patients, suggesting that the decrease in frontal cortical executive function might be an independent accompanying factor for OAB and could influence the severity of storage symptoms in PD patients. Previous animal studies have demonstrated that the frontal cortex, and especially the prefrontal cortex, plays a significant role in inhibiting the micturition reflex during the storage phase [19]. Haruta et al. found that performance on the FAB inhibitory control task is decreased in vascular incontinence patients with detrusor overactivity, which implied that the bladder is under general inhibitory control concerning decision-making and emotion by the prefrontal cortex [20]. Functional brain imaging studies have revealed a frontal deactivation in the women with urge incontinence as compared with controls [21, 22]. The pathogenesis of this dysfunction might be that the frontal lobe can no longer adequately inhibit the micturition reflex, leading to increases in urinary frequency, urinary incontinence, and other symptoms of overactive bladder, which can be regarded as an exaggerated micturition reflex [23].

When compared with functional neuroimaging, the FAB scale [16], which was devised by Dubois et al. is easier to administer, because the evaluation can be performed at bedside. Furthermore, the FAB scale is a sensitive tool for monitoring frontal lobe dysfunction. Therefore, the FAB scale can be used to evaluate patients who complain about LUT symptoms and determine whether their LUT dysfunction is caused by impaired frontal lobe function. If it is, the patient's frontal lobe function should be improved in a timely manner in order to prevent any further aggravation of the LUT symptoms.

A comparative analysis of clinical data for the patients in our study confirmed that the mean H-Y stage of patients in the PD-OAB group was significantly higher than that of patients in the PD-NOAB group and identified the H-Y stage as an accompanying factor for OAB in PD. We also found that increases in the H-Y stage corresponded with increases in the OABSSs, suggesting that the LUT symptoms in PD patients may be closely correlated with nigral dopaminergic depletion. Our findings are in accordance with those reported by Mito et al. [24], who found that higher OABSSs were consistently associated with an increase in the severity of motor disorders. The reason for that correlation may be that a reduction of dopamine levels in the nigrostriatal leads to a decrease in micturition reflex inhibition.

Previous functional neuroimaging studies revealed a significant decrease of dopamine transporter imaging in the brains of PD patients with urinary dysfunction [25, 26]. Animal studies have confirmed that dopamine secreted by the substantia nigra pars compacta suppresses the micturition reflex via the dopamine D1 receptor and that a loss of dopaminergic neurons can cause detrusor hyperreflexia [27]. Brusa et al. found that acute levodopa therapy stimulates predominantly D2 receptors, which may result in exacerbation of storage dysfunction. However, chronic levodopa therapy stimulates both D1 and D2 receptors, which may lead to an improvement of LUT function [28]. Therefore, we hypothesize that timely treatment with levodopa and a partial agonist at dopamine D1 receptors in patients with clinically diagnosed early PD can not only improve those patients' motor symptoms but can also help prevent the occurrence of OAB symptoms and the progression of LUT dysfunction. Although our results did not find that an equivalent dose of levodopa helps to protect against OAB, this may be related to the small sample size of our study and needs to be investigated in the future.

A previous study showed that nigral dopaminergic depletion is related to akinesia/rigidity, but not tremor [29]. Even though our study found no relationship between OABSSs and different PD subtypes, we cannot rule out that the influence of parkinsonian tremor might be somewhat different from those of akinesia/rigidity. Future studies should evaluate the possible effects of those factors.

Several specific nonmotor symptoms are also known to be associated with LUT dysfunction [30, 31]. Our results suggested a possible connection between RBD and LUT dysfunction in PD. On the one hand, this may be due to the locus coeruleus and pontine nucleus, which are related to RBD, overlap with some regions that control micturition. On the other hand, it may be correlated with a reduction of inhibitory neurotransmitters in the brain, such as GABA and 5-HT [32].

The pontine micturition center is located on the ventral medial side of the pontine storage center, adjacent to the locus coeruleus, which can project spinal fibers containing excitatory and inhibitory neurotransmitters downward [23]. Therefore, when *α*-synuclein is deposited in the locus coeruleus, RBD will appear. When *α*-synuclein becomes deposited in the pontine micturition center, the transmission of inhibitive neurotransmitters in the body decreases and symptoms of OAB will appear or become aggravated. Previous studies have reported that benzodiazepine drugs, anticholinergic drugs, or norepinephrine and 5-HT reuptake inhibitors may be effective for treating LUT symptoms [33, 34]. Zoetmulder et al. found that dopamine function may have an effect on increased motor activity during REM sleep in PD patients with RBD [35]. In addition, dopamine D2-receptor immunoreactivity has been found in brainstem structures involved in REM sleep without atonia [36, 37]. Therefore, we hypothesize that the decrease of dopamine in PD patients' brain will lead to the decreased stimulation of D1 and D2 receptors by direct and indirect pathways, which will produce OAB and RBD, respectively. The pathological and pathophysiological mechanisms of RBD and LUT dysfunction lead one to speculate about a possible clinical correlation between those two disorders.

The possible correlation between age and the presence of LUT symptoms in PD patients has been previously studied. Some studies reported correlations among them [38, 39], while others failed to find any correlation [40]. In this study, which employed relatively strict patient selection criteria, we also found a correlation between age and the presence of LUT symptoms. This finding was in agreement with Campos-Sousa et al., who reported that the age of patients in a control group was correlated with urinary dysfunction [38]. However, a logistic regression analysis showed that age was just a confounding factor. This reinforces evidence that LUT dysfunction is age-associated and not an age effect [41]. Therefore, in clinical practice, it is necessary to pay attention to the LUT symptoms of older PD patients and determine whether they require further treatment.

Our study did not find any relationship between a patient's OABSS and sex, disease duration, or education. Previous studies have failed to reach a consensus on this topic [38, 42, 43]. The reason for this might be very complicated. In our cohort, we accidently found that LUT symptoms mostly appear after PD onset and that their occurrence increases in conjunction with disease duration. Because it is well known that micturition disorders in multiple system atrophy (MSA) patients usually occur during the early stage of their disease, or even prior to MSA onset, those LUT disorders might be useful as biological indicators for identifying PD and MSA.

Our study has limitations. First, this is only a cross-sectional design with no causal inference, and it needs further studies to confirm the results. Secondly, this is a single-center trail with small number of cases. It is better to expand the sample size and design multicenter test for further research. Furthermore, our study lacks information of some other important nonmotor features such as orthostatic hypotension, and further evaluation will be required.

## 5. Conclusions

In summary, our study showed that decreased frontal lobe executive function, increased severity of motor disorders, and RBD are independent accompanying factors for OAB. An aggravation of LUT dysfunction in PD patients was consistently associated with the above three factors and also old age. This association may be related to decreased inhibition of the micturition reflex by the superior central nervous system and a neurotransmitter imbalance, which leads to detrusor hyperreflexia. We believe that our research may help to improve the clinical diagnosis and treatment of PD, identify its underlying pathogenesis, and assist in establishing a prognosis for PD patients.

## Figures and Tables

**Table 1:** Demographic and clinical characteristics of the enrolled PD patients and the univariate analysis between PD-OAB and PD-NOAB.

	Enrolled PD patients	PD-OAB	PD-NOAB	*t*/*χ* ^2^/*Z*	*P*
Number of patients	100	45 (45%)	55 (55%)		
Gender (male/female)	55/45	29/16	26/29	2.949	0.086
*Age* (*years*)	65.97 ± 8.247 (range 48–85)	69.36 ± 7.53	63.20 ± 7.82	−3.982	**<0.001** ^*∗*^
*Age of onset* (*years*)	61.45 ± 8.361 (range 40–81)	64.30 ± 7.83	59.13 ± 8.12	−3.217	**0.002** ^*∗*^
Disease duration (years)	4 (2, 7) (range 0.25–20)	4 (2, 7)	3.5 (1, 6)	−0.984	0.325
Education (years)	9 (9, 12) (range 0–20)	9 (9, 12)	10 (9, 12)	−0.774	0.439
LEDD (mg)	350 (178.13, 562.50) (range 0–1425)	337.5 (193.75, 668.75)	375 (150, 525)	−0.347	0.728
Motor symptom					
*Hoehn–Yahr stage*	2 (2, 3) (range 1–5)	2.5 (2, 3.5)	2 (2, 2)	−2.851	**0.004** ^*∗*^
*MDS-UPDRS-III score*	35 (27, 47.75) (range 12–112)	39 (28.5, 56)	34 (24, 44)	−2.069	**0.039** ^*∗*^
Clinical subtype					
Tremor-dominant (yes/no)	67/33	30/15	37/18	0.004	0.949
Akinetic/rigid-dominant (yes/no)	44/56	20/25	24/31	0.007	0.935
Nonmotor symptom					
Hyposmia (yes/no)	39/61	19/26	20/35	0.357	0.550
*RBD* (*yes/no*)	58/42	31/14	27/28	3.982	**0.046** ^*∗*^
Constipation (yes/no)	72/28	33/12	39/16	0.072	0.788
Anxiety and depression (yes/no)	55/45	26/19	29/26	0.255	0.614
Hallucination (yes/no)	11/89	6/39	5/50	0.455	0.536
MMSE score	28 (26, 29) (range 11–30)	27 (25.5, 29)	28 (26, 29)	−1.454	0.146
*MoCA score*	24 (21.25, 27) (range 7–30)	24 (20, 26)	26 (22, 28)	−2.721	**0.007** ^*∗*^
*FAB score*	15 (14, 16) (range 6–18)	14 (12, 15)	16 (15, 17)	−5.034	**<0.001** ^*∗*^

^*∗*^ *P* < 0.05; the difference was statistically significant.

**Table 2:** Risk factors for OAB.

Factor	*B*	S.E.	Wald	OR	95% CI of *B*		*P* value
					Lower	Upper	
Hoehn–Yahr stage	1.101	0.400	7.577	3.007	1.373	6.585	**0.006** ^*∗*^
RBD	1.228	0.535	5.264	3.414	1.196	9.745	**0.022** ^*∗*^
FAB score	1.207	0.304	15.726	3.344	1.842	6.074	**<0.001** ^*∗*^

^*∗*^ *P* < 0.05; the difference was statistically significant.

**Table 3:** Multiple linear regression coefficients of OABSS score.

Items	*B*	S.E.	95% CI of *B*		*t*	*P* value
			Lower	Upper		
Age (years)	0.125	0.042	0.042	0.207	2.992	**0.004** ^*∗*^
Hoehn–Yahr stage	1.068	0.479	0.117	2.018	2.231	**0.028** ^*∗*^
RBD	1.553	0.608	0.347	2.759	2.556	**0.012** ^*∗*^
FAB score	1.252	0.288	0.680	1.824	4.347	**<0.001** ^*∗*^
Constant	−8.644	2.518	−13.643	−3.646	−3.433	0.001

^*∗*^ *P* < 0.05; the difference was statistically significant.

## Data Availability

The underlying data related to this study are available from the corresponding author upon request but not for the commercial activities.

## Supplementary Materials

Supplementary Materials The questionnaire used to define hyposmia, RBD, constipation, anxiety, depression and hallucination.
