# Supplementary material for: Relationship between Lower Urinary Tract Dysfunction and Clinical Features in Chinese Parkinson's Disease Patients
Source: Parkinsons Dis. 2019 Mar 5;2019:6820937. doi: 10.1155/2019/6820937 (PMC6425341; doi:10.1155/2019/6820937)
Supplement: Supplementary Materials — The questionnaire used to define hyposmia, RBD, constipation, anxiety, depression and hallucination. [file 6820937.f1.pdf]

PD NMS QUESTIONNAIRE

Name: .... Date: .... Age: ....

Centre ID: Male ☐ Female ☐

NON-MOVEMENT PROBLEMS IN PARKINSON'S

The movement symptoms of Parkinson's are well known. However, other problems can sometimes occur as part of the condition or its treatment. It is important that the doctor knows about these, particularly if they are troublesome for you.

A range of problems is listed below. Please tick the box 'Yes' if you have experienced it **during the past month.** The doctor or nurse may ask you some questions to help decide. If you have **not** experienced the problem in the past month tick the 'No' box. You should answer 'No' even if you have had the problem in the past but not in the past month.

Have you experienced any of the following in the last month?

	Yes	No
**1.** Dribbling of saliva during the daytime	☐	☐
**2.** Loss or change in your ability to taste or smell	☐	☐
**3.** Difficulty swallowing food or drink or problems with choking	☐	☐
**4.** Vomiting or feelings of sickness (nausea)	☐	☐
**5.** Constipation (less than 3 bowel movements a week) or having to strain to pass a stool (faeces)	☐	☐
**6.** Bowel (fecal) incontinence	☐	☐
**7.** Feeling that your bowel emptying is incomplete after having been to the toilet	☐	☐
**8.** A sense of urgency to pass urine makes you rush to the toilet	☐	☐
**9.** Getting up regularly at night to pass urine	☐	☐
**10.** Unexplained pains (not due to known conditions such as arthritis)	☐	☐
**11.** Unexplained change in weight (not due to change in diet)	☐	☐
**12.** Problems remembering things that have happened recently or forgetting to do things	☐	☐
**13.** Loss of interest in what is happening around you or doing things	☐	☐
**14.** Seeing or hearing things that you know or are told are not there	☐	☐
**15.** Difficulty concentrating or staying focussed	☐	☐
**16.** Feeling sad, 'low' or 'blue'	☐	☐
**17.** Feeling anxious, frightened or panicky	☐	☐
**18.** Feeling less interested in sex or more interested in sex	☐	☐
**19.** Finding it difficult to have sex when you try	☐	☐
**20.** Feeling light headed, dizzy or weak standing from sitting or lying	☐	☐
**21.** Falling	☐	☐
**22.** Finding it difficult to stay awake during activities such as working, driving or eating	☐	☐
**23.** Difficulty getting to sleep at night or staying asleep at night	☐	☐
**24.** Intense, vivid dreams or frightening dreams	☐	☐
**25.** Talking or moving about in your sleep as if you are 'acting' out a dream	☐	☐
**26.** Unpleasant sensations in your legs at night or while resting, and a feeling that you need to move	☐	☐
**27.** Swelling of your legs	☐	☐
**28.** Excessive sweating	☐	☐
**29.** Double vision	☐	☐
**30.** Believing things are happening to you that other people say are not true	☐	☐

All the information you supply through this form will be treated with confidence and will only be used for the purpose for which it has been collected. Information supplied will be used for monitoring purposes. Your personal data will be processed and held in accordance with the Data Protection Act 1998.

Developed and validated by the International PD Non Motor Group
For information contact: susanne.tluk@uhl.nhs.uk or alison.forbes@uhl.nhs.uk
